# A case study of pyloric stenosis caused by metastatic lobular carcinoma of breast

**DOI:** 10.1093/jscr/rjad691

**Published:** 2023-12-28

**Authors:** Taizo Yoshida, Akihiko Shimada, Koichi Matsumoto, Michiko Inukai, Ryo Hashimoto, Mutsuhito Matsuda, Tomohiko Nishi, Hiroaki Seki, Michio Sakata, Hidetoshi Matsumoto

**Affiliations:** Department of Surgery, Keiyu Hospital, 3-7-3 Minatomirai, Nishi-ku, Yokohama, Kanagawa 220-8521, Japan; Department of Surgery, Keiyu Hospital, 3-7-3 Minatomirai, Nishi-ku, Yokohama, Kanagawa 220-8521, Japan; Department of Surgery, Keiyu Hospital, 3-7-3 Minatomirai, Nishi-ku, Yokohama, Kanagawa 220-8521, Japan; Department of Surgery, Keiyu Hospital, 3-7-3 Minatomirai, Nishi-ku, Yokohama, Kanagawa 220-8521, Japan; Department of Surgery, Keiyu Hospital, 3-7-3 Minatomirai, Nishi-ku, Yokohama, Kanagawa 220-8521, Japan; Department of Surgery, Keiyu Hospital, 3-7-3 Minatomirai, Nishi-ku, Yokohama, Kanagawa 220-8521, Japan; Department of Surgery, Keiyu Hospital, 3-7-3 Minatomirai, Nishi-ku, Yokohama, Kanagawa 220-8521, Japan; Department of Surgery, Keiyu Hospital, 3-7-3 Minatomirai, Nishi-ku, Yokohama, Kanagawa 220-8521, Japan; Department of Surgery, Keiyu Hospital, 3-7-3 Minatomirai, Nishi-ku, Yokohama, Kanagawa 220-8521, Japan; Department of Surgery, Keiyu Hospital, 3-7-3 Minatomirai, Nishi-ku, Yokohama, Kanagawa 220-8521, Japan

**Keywords:** gastrointestinal metastasis, pyloric stenosis, invasive lobular carcinoma of the breast, immunohistochemical analysis

## Abstract

Metastasis to the gastrointestinal tract is rare. A 59-year-old woman who had a history of an invasive lobular carcinoma of breast with clinical complete response visited our hospital and complained of an upper abdominal pain and distension. We performed an upper gastrointestinal endoscopy which showed only a gastric ulcer without any malignant findings. She experienced a recurrence of symptoms 2 months after this visit. An endoscopy revealed pyloric stenosis, which did not improve with balloon dilatation. We performed a gastro-jejunal and cecal-transverse colonic bypass surgery. Diffuse wall thickening of the antrum was verified during the surgery, and a biopsy sample was collected. The diagnosis of gastric metastasis from breast was confirmed since it showed the same immunohistochemistry pattern as the prior breast lesion. Pyloric stenosis has still been confirmed with an endoscopy, she has been alive with satisfactory oral food intake for >10 years.

## Introduction

Breast cancer is one of the most diagnosed cancers in the world and is accounted for 11.7% of all cancer cases in female [1]. Lungs, bones, soft tissues, and livers are common sites of metastasis. Till date, only few cases of metastasis to the gastrointestinal (GI) tract are reported [2, 3].

In the present study, we described a woman with breast cancer who experienced upper abdominal discomfort and distention as a result of gastric metastasis. The upper GI endoscopy was performed several times, but no malignant biopsy results were observed until bypass surgery. This case made people more aware of gastric metastases from the breast that does not show any malignant findings on imaging tests.

## Case report

**Figure 1:**
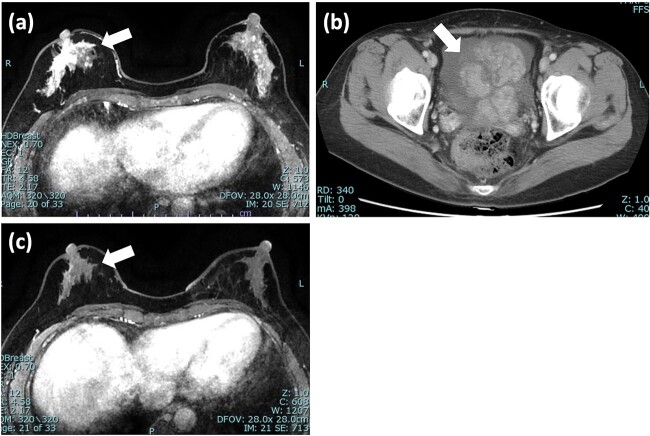
Imaging tests of right breast cancer and ovarian tumor. (a) MRI of the breast showed heterogeneous enhanced lesion below the nipple; (b) CT scan of pelvis revealed an ovarian tumor with increased ascites; (c) MRI after 5 months showed significant reduction in the size of tumor and diminished enhancement. MRI: magnetic resonance imaging; CT: computed tomography.

**Figure 2:**
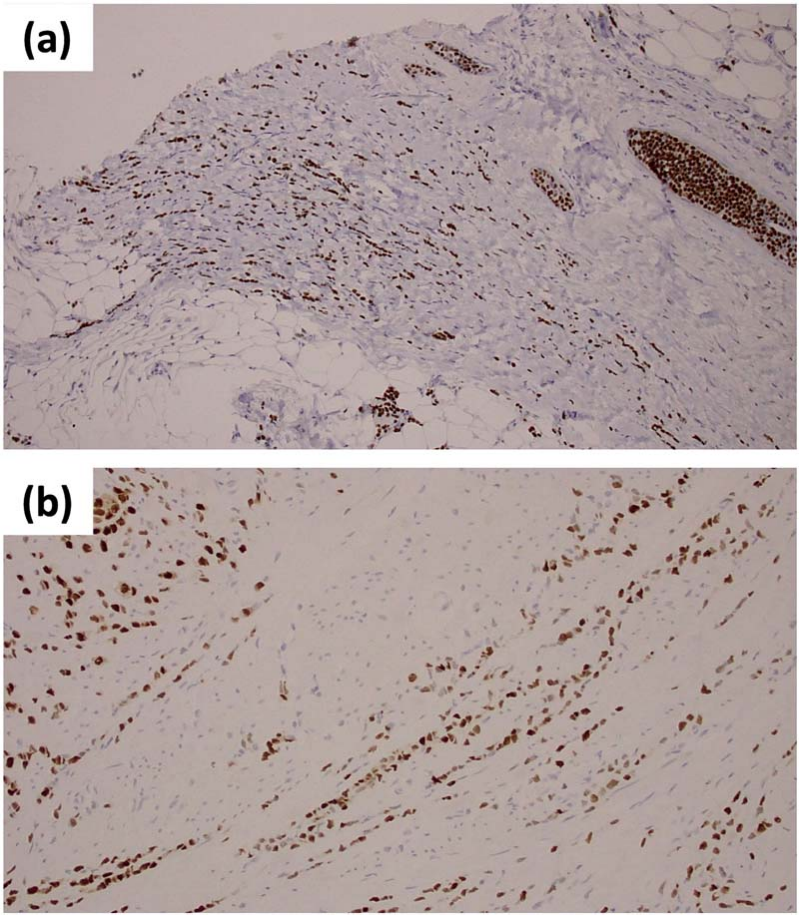
Immunohistochemical staining of ER (a) the primary breast lesion; (b) biopsy samples from the anterior wall of gastric antrum.

A 55-year-old woman was diagnosed with invasive lobular carcinoma (ILC) in her right breast (Fig. 1a). Breast biopsy revealed Luminal A subtype with positive estrogen-receptor (ER), progesterone-receptor (PgR), negative HER2 expression, and low Ki-67 index (5%) (Fig. 2a). She received neoadjuvant chemotherapy, which comprises two cycles of epirubicin plus cyclophosphamide (EC) therapy. However, a left salpingo-oophorectomy was carried out after a computed tomography (CT) scan identified an ovarian tumor, which was assumed to represent the primary disease (Fig. 1b). She was diagnosed with ovarian metastasis from breast cancer after histopathological samples of her left ovary revealed the same pattern as the breast cancer. She was treated with chemotherapy (docetaxel; DTX) and hormonal therapy (anastrozole), which led to a clinical complete response (cCR) for the breast cancer lesion (Fig. 1c).

**Figure 3:**
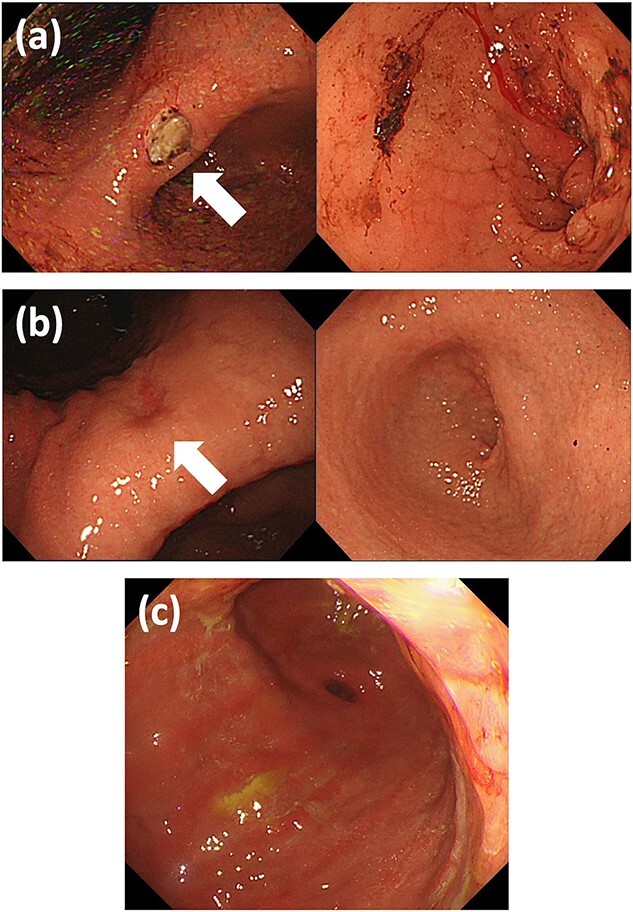
Upper GI endoscopy findings (a) at the first visit: ulcer at the gastric angle without pyloric stenosis; (b) when the symptoms recurred: healed gastric ulcer and diffusely thickened stenotic pyloric ring; and (c) after the bypass surgery: diffuse infiltration of tumor at the antrum. GI: gastrointestinal.

The patient visited our hospital 4 years later, at the age of 59, complaining of upper abdominal discomfort and distention. An upper GI endoscopy revealed an ulcer at the angle of the stomach but could not confirm any stenotic lesions or gastric tumor (Fig. 3a). Her symptoms improved with a nasogastric tube placement. However, her symptoms reappeared 2 months later and we performed upper GI endoscopy again which showed diffuse wall thickening at the antrum without any gross signs of tumor (Fig. 3b). This lesion’s histopathological investigation revealed no indications of cancer. Although we repeatedly conducted endoscopic balloon dilatation of the pyloric ring, her problem was persisted. Therefore, we performed a gastro-jejunal bypass surgery.

During the procedure, we confirmed the diffuse solid change in the antrum wall suggestive of gastric tumor which might have invaded transverse colon. Therefore, in addition to a gastro-jejunal bypass, we performed a cecal-transverse colonic bypass surgery. We collected biopsy samples from the anterior wall of the antrum and the solid lesion from retroperitoneum. Both samples revealed the formation of intracytoplasmic lumens and tumor cells organized in trabecular patterns. The biopsy samples from this patient’s prior breast surgery showed ILC with the same histological pattern as the gastric sample, suggesting that the breast cancer had spread to the stomach. Immunohistochemical analysis showed tumor cells which were positive for ER and PgR (Fig. 2b). These findings confirmed the diagnosis of gastric metastasis from the breast cancer.

The patient received fulvestrant and palbociclib treatment together with toremifene for 2 years. She has been alive with cCR for the breast lesion for >10 years with a satisfactory oral food intake, although the metastatic gastric lesion still exists (Fig. 3c).

## Discussion

Breast cancer is the most common disease in female today and frequent sites of metastasis are reported to be lungs, bones, soft tissues, and livers [1]. However, metastatic disease to GI tract is rare, which is reported in only 41 cases out of 12 001 cases diagnosed with metastatic disease secondary to breast cancer [2]. It has been classified into different categories such as lobular and ductal carcinoma. An increased risk of secondary gastric cancer occurs in ILC patients, and there was 84% increased incidence of gastric cancer were reported after the ILC diagnosis [4].

ILC showed a different metastatic pattern than invasive ductal carcinoma. ILC was more likely to involve the GI tract, and upper GI metastases are more frequently reported than lower GI metastases in ILC patients [5]. This suggests that it is essential to consider metastases if an ILC patient complains of upper GI symptoms.

In the present case, malignant findings were not observed during the upper GI endoscopy, which suggested that the upper GI endoscopy was not sensitive enough to detect gastric cancer that had spread from breast. In a previous report, 10 out of 51 cases of breast cancer patients with gastric metastasis showed negative by upper GI endoscopy [6]. All layers of the stomach, excluding the mucosa, are often involved in the typical metastatic pattern of ILC in stomach [7], which may make it hard to detect gastric metastasis. Tall *et al.* [6] described three types of endoscopic features of metastatic gastric tumor: localized tumor deposition with ulceration, diffuse infiltration, and external compression. The most frequent type was diffuse infiltration pattern, which was observed in the present case. When we perform upper GI endoscopy in patients with a history of ILC of the breast, we always need to be cautious that negative biopsy results from upper GI endoscopy cannot exclude breast cancer metastasis.

In ILC patients with gastric metastasis, a careful assessment of histopathological examination is required. ILC metastatic and primary gastric cancer are difficult to distinguish from each other because metastatic gastric cancer mimics signet ring cells [4]. Immunohistochemical marker is an important diagnostic technique for gastric metastasis from the breast cancer. However, it has been observed that HER2 and hormone receptors are expressed in primary gastric cancer [8, 9], by comparing them with the biopsy samples collected from the breast cancer, the combination of these expressions might be extremely important in the final diagnosis. Our biopsy results showed the same immunohistochemical pattern as the previous breast lesion which concluded the diagnosis. Although we only investigated the expression of ER and PgR, gross cystic disease fluid protein 15 (GCDFP-15) and GATA binding protein 3 (GATA3) are also reported to be helpful for differential diagnosis for metastatic breast cancer [10].
